# The genetic composition of Shina population from Gilgit-Baltistan, Pakistan based on mtDNA analyses

**DOI:** 10.1080/23802359.2019.1682474

**Published:** 2019-10-26

**Authors:** Mah Noor Mumtaz, Haleema Ihsan, Shahid Aziz, Hizbullah, Sahib Gul Afridi, Sulaiman Shams, Asifullah Khan

**Affiliations:** Department of Biochemistry, Abdul Wali Khan University, Mardan 23200, Khyber Pakhtunkhwa, Pakistan

**Keywords:** Population genetics, mitochondrial DNA, Shina population, Gilgit-Baltistan Pakistan

## Abstract

The present study aimed to gain insight into the genetic origin of the Shina population from Gilgit-Baltistan, Pakistan. We partially performed the mitochondrial DNA (mtDNA) control region of healthy unrelated individuals of Shina tribe residing in the remote area of Gilgit-Baltistan to investigate their maternal lineages. The present study is the first report about Shina’s genetic structure, origin, and relationship with the surrounding north-western Pakistani population. The mtDNA sequences of the Shina samples were compared with the revised Cambridge Reference Sequence (rCRS) and the HVR-1 D-loop region was covered. The comparison with rCRS identified overall 38 haplotypes and 08 haplogroups for Shina samples. Among these haplotypes, 18 were shared by more than one individual of the Shina tribe. The obtained mtDNA sequences of Shina were compared with surrounding north-western Pakistani population groups, i.e. Kho, Kashmiri, and Pathan. The genetic diversity (i.e. 1.0424) and power of discrimination (i.e. 0.9266) of the Shina was found equivalent to surrounding north-western groups. The haplogroups frequencies, phylogenetic tree and network analysis identified the west Eurasian ancestral origin of Shina group with nearby maternal ancestral relationships with the Kashmiri population. However, no close genetic relationship of Shina was depicted with nearby residing Kho population group.

## Introduction

Pakistan is located at the crossroads of Asia on the intersection of the Middle East, Central Asia, and Southeast Asia. The early human migration from Africa to South Asia and Australia occurred across the Pakistani corridor around 50,000–70,000 years ago (Petraglia et al. 2012). Pakistani population is comprised of 16 small and large ethnic groups on the basis of different languages and cultures (Ayub and Tyler-Smith 2009). About 3000 years before present (YBP) the Fertile Crescent (the area between Southeast Anatolia and Zagros mountains) had history of urban civilization and served as a passage for human migration between Mesopotamia and Iranian Plateau including the north-western part of Pakistan (Di Cristofaro et al. 2013). A previous report revealed that the population situated in the west of Pakistan mainly consists of western Eurasian mtDNA lineages with a restricted South Asian contribution (Bhatti et al. 2017).

The Gilgit-Baltistan is the northernmost administrative unit of Pakistan formerly called as ‘northern regions’. The region is highly mountainous and divided into three parts Gilgit, Diamer, and Baltistan covering an area of 72,496 km^2^. The recorded population of Gilgit-Baltistan is 1.4 million as according to the 2017 population census. Geographically, the region shares the borders with Pakistan's Azad Jammu and Kashmir in the south and meets the Khyber Pakhtunkhwa province in the west, a small part of Afghanistan's Wakhan corridor to the north, Xinjiang (China) from the east and north-east and India-administered Kashmir to the southeast. The linguistic groups of Gilgit-Baltistan are Shina, Balti, Wakhi, Domaki, Khowar, Burushaski and Gojri (Khan 2017).

**Figure 1.**
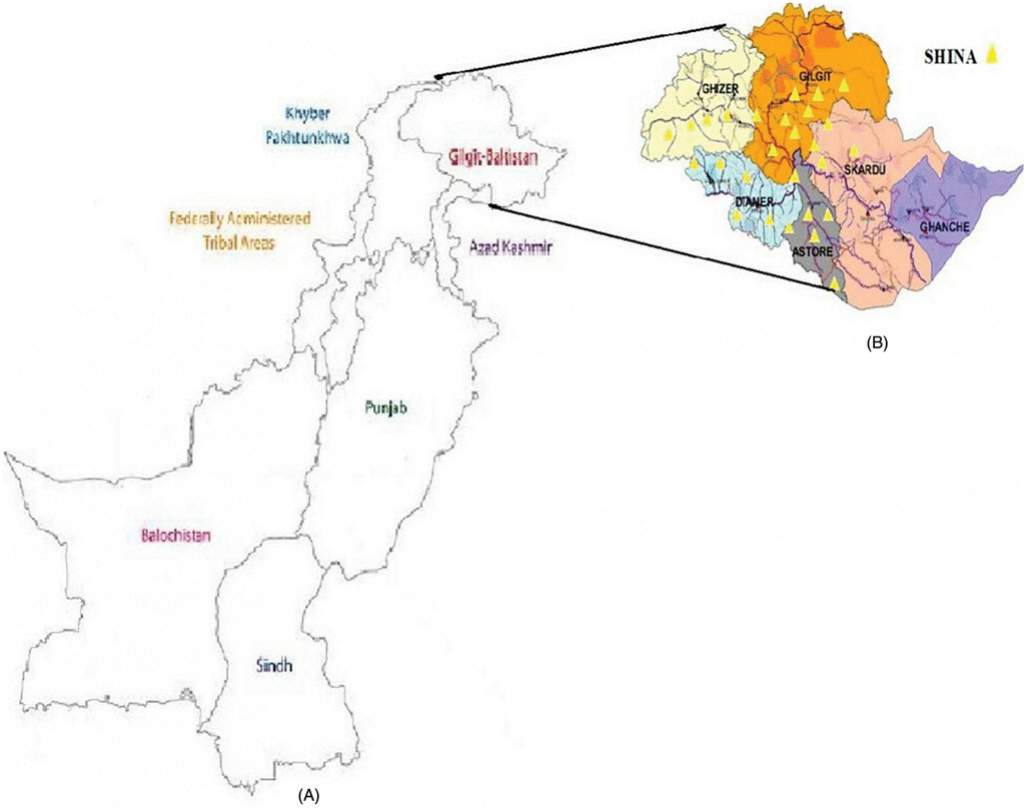
(A) Map of Pakistan presenting Gilgit-Baltistan. (B) Detail map of the studied area, i.e. Shina population from Gilgit-Baltistan. Yellow arrows represent Shina tribe residing in different districts of Gilgit-Baltistan.

The Shina people also known as shin migrated from Central Asia to South Asia during the first half of the second millennium BC. They live in Southern Gilgit-Baltistan, Chitral and the western part of the Kohistan region of Khyber Pakhtunkhwa, Pakistan (Figure 1). The Shina people reside in Gilgit, Ghizer, Hunza, Nagar, Diamer, Ghanche, Skardu, Danyore and Astore districts of Gilgit-Baltistan. They also live in the Kishenganga and Dras valley in northern Jammu and Kashmir, India. Shina is one out of the 16 ethnic groups of the Pakistani population (Ayub and Tyler-Smith 2009). About five to seven thousand years ago, the early entrance model suggests that the ancestors of Shina and other residents of northern Pakistan were subjected to gene flow from Central Asia to South Asia due to the migration and earlier settlement. Hence, Shina people hold similarities to prehistoric Central Asians, living in north-west India and in the Indus Valley. According to some reports, the Shina individuals entered to Gilgit-Baltistan and the surrounding area in 1680 (Hemphill 2012). The Shina people speak Dardic language called Shin which is a subset of the Indo-Aryan languages brought to South Asia by the Central Asian invaders. The Shina is the major ethnic group of Gilgit-Baltistan and Shin language is spoken by an estimated 500,000 people living mainly in Gilgit, Diamer, and Baltistan of Gilgit-Baltistan autonomous region and Kohistan District of the Khyber Pakhtunkhwa province of Pakistan. The Shina individuals differ from other Pakistani people on the basis of a different culture, norm, and language (Kachru et al. 2008).

Human mtDNA has been studied to investigate the evolution and genetic origin of human populations (Lee et al. 2013). The D-loop (displacement loop) region or control region is the large noncoding segment in the mitochondrial genome. The polymorphism in the D-loop region of mtDNA is important to decipher the population maternal lineages (Behar et al. 2008). The present study was carried out to identify the genetic pattern of the mtDNA D-loop region of the Shina population from Gilgit-Baltistan to characterize its maternal lineages. Furthermore, comparative genetic analyses of Shina samples with surrounding population groups were performed to understand its genetic relationship in context with nearby residing north-western Pakistani population groups.

## Materials and methods

### Blood sampling and DNA extraction

About 3 ml blood samples were collected from healthy unrelated 07 male and 02 female individuals of Shina tribe belonging from Gilgit-Baltistan (Supplementary Material; Table S1). Informed consent was obtained from all the study participants. The collected blood samples were preserved in EDTA vacutainers. DNA was extracted from collected blood samples according to the published phenol/chloroform method (Kochl et al. 2005). The quality and quantity of genomic DNA were checked by agarose gel electrophoresis observed under UV transilluminator of a gel documentation system (Supplementary Material; Figure S1).

### PCR amplification and mtDNA sequencing

A set of primers reported by Hayat et al. (2015) were used for PCR amplification of target mtDNA HVRs region comprises of 1122 bp. The genomic DNA of about 30–40 ng and both forward and reverse primers of 1–2 ng were used during the PCR reaction. The PCR reaction was performed in 30 μl total volume using Ampi Taq Gold Master Mix (Cat.no. REF K1081, LOT-00640658) under the conditions; Initial denaturation at 96 °C for 5 minutes, followed by 34 cycles at 96 °C for 5 minutes, annealing at 60 °C for 45 seconds, extension at 72 °C for 1 minute and final extension at 72 °C for 5 minutes. The DNA sequencing of PCR amplified products was carried out based on Sanger method using ABI 3130 genetic analyzer. The forward primer (i.e. F15975) used during PCR reaction was reused for cycle sequencing chain termination reaction.

### Data analyses

#### mtDNA sequences quality check and comparative population genetics analysis

The DNA sequence reads trace quality check was performed using Staden package (Staden et al. 2000) and Lasergene v. 7. 1 (DNASTAR Inc., Madison, WI, USA). The good quality sequence reads in triplicates were assembled via DNASTAR's SeqMan and high-quality contigs were obtained. The DnaSP 5.10 software was used to evaluate mutations and parsimony informative sites (Rozas et al. 2017). The mtDNA sequences of other north-western Pakistani populations (i.e. Kashmiri, Pathan, and Kho) were obtained from Genbank, National Centre for Biotechnology Information (NCBI) (http://www.ncbi.nlm.nih.gov) (Supplementary Material; Table S2). These sequences were trimmed according to Shina high-quality mtDNA sequences to perform comparative population genetic analyses.

#### Haplotypes and haplogroup analysis

The haplotypes and haplogroups prediction from sequences was performed using MITOMASTER bioinformatics tool which makes an accurate analysis to identify the haplogroups and haplotypes from human mtDNA sequences (Brandon et al. 2009).

#### Phylogenetic analysis

The phylogenetic tree of Shina mtDNA sequences in comparison with surrounding populations (Kashmir, Pathan, and Kho) was constructed using MEGA7.0.9 software. The neighbour-Joining (NJ) and maximum-likelihood (ML) methods were followed with 10,000 bootstrap replicates during this analysis (Kumar et al. 2016).

#### Haplotypes network analysis

The haplotypes network analyses were performed through NETWORK 5.0 software (Gehring et al. 2012). The median-joining method processed mainly by star contraction (SC) and maximum parsimony (MP) calculations were followed. The star radius parameter in SC was set as 5. The MP calculated data then subjected to networking analysis. A network plot was obtained where population groups were indicated by a different colour.

#### Access to data

The mtDNA sequences of Shina samples were submitted to Genbank, NCBI under the accession numbers; MK805520-MK805528.

## Results

The mtDNA hypervariable region of 09 unrelated samples from Shina individuals was sequenced to investigate their maternal lineage and genetic structure. All the samples were sequenced in triplicate (3×) to confirm the sequence variants. The high-quality nucleotide draft sequences of 504 bp were generated covering the 16,066–16,569 positions of the rCRS mtDNA D-loop region (HVR-1). The mtDNA sequences were downstream analyzed for possible mtDNA haplotypes and haplogroup composition. A total of 38 mtDNA haplotypes were identified for Shina’s mtDNA sequences among which 18 were shared by more than one individual. The haplotypes based on C16073A (18.42%) and T16519C (13.15%) SNPs were found frequently. Besides, 08 different haplogroups were observed for Shina’s mtDNA sequences. A haplogroup H2a was found shared in Shina samples (Table 1). In comparison with rCRS, 85% of transition mutations were found at 23 different positions, while 15% of transversion mutations were found at 4 different sites. No deletion and insertion mutations were identified for Shina samples sequences (Supplementary Material; Table S3). The West Eurasian haplogroups were most commonly observed in Shina samples with a total frequency of 89%. These include the H14a (11.1%), T1a (11.1%), H2a (22.2%), T2 (11.1%), U7 (11.1%), U5b (11.1%) and HV2 (11.1%) haplogroups (Table 2). Besides, the West Eurasian, a M54 haplogroup of South Asian origin (Maji et al. 2009; Chandrasekar et al. 2009) was also detected at 11.1% frequency in Shina samples.

**Table 1.** The haplotypes and haplogroups identified for Shina population samples.

Sample ID	HG	HG O	Haplotype
S-1	H14a	West Eurasian	**C16073A**, C16256T, T16352C, T16422C
S-2	M54	South Asian	**C16073A**, C16223T, C16266T, T16304C, C16446T, **T16519C**
S-3	T1a	West Eurasian	**T16126C**, A16163G, C16186T, T16189C, **C16294T**, **T16519C**
S-4	H2a	West Eurasian	**C16073A**, **T16519C**
S-5	T2	West Eurasian	**C16073A**, **T16126C**, **C16294T**, C16296T, T16325C, G16412C, **G16496C**, **T16519C**, C16527T, G16558A
S-6	H2a	West Eurasian	**C16073A**
S-7	U7	West Eurasian/South Asian	**C16073A**, A16309G, A16318T
S-8	U5b	West Eurasian	**C16073A**, **G16496C**, G16526A, G16543A
S-9	HV2	West Eurasian	T16217C, **T16519C**

The bold character in table indicates haplotypes shared among Shina individuals. S: Shina; HG: haplogroup; HG O: haplogroup origin.

**Table 2.** The mtDNA haplogroups identified for Shina samples.

Haplogroup	Sample	Frequency (%)	Haplogroup	Sample	Frequency (%)
H14a	S-1	11.1	T2	S-5	11.1
M54	S-2	11.1	U7	S-7	11.1
T1a	S-3	11.1	U5b	S-8	11.1
H2a	S-4, S-6	22.2	HV2	S-9	11.1

S: Shina.

We performed an additional comparative analysis of the Shina mtDNA sequences to know their maternal lineage relationship with closely residing north-western Pakistani populations (i.e. Kashmiri, Pathan, and Kho). The mtDNA HVR sequences of these population were acquired from GenBank, NCBI database. The Shina samples were found to have few shared haplogroups and haplotypes with surrounding northwestern Pakistani populations (Table 3). The basic population genetic statistical parameters including the genetic diversity, random match probability and power of discrimination of Shina in comparison with other north western population are given in Table 4.

**Table 3.** The shared haplogroups and haplotypes of Shina with other north-western Pakistan populations.

Shina population`s unique		Shina & Kashmiri population shared		Shina & Pathan population shared		Shina & Kho population shared	
Haplogroups	Haplotypes	Haplogroups	Haplotypes	Haplogroups	Haplotypes	Haplogroups	Haplotypes
H14a	C16073A	H2a	T16189C	HV2	C16223T		C16223T
M54	C16446T		T16519C		T16217C	0	T16519C
T1a	T16352C	U7	C16223T		T16126C		T16189C
T2	T16422C		T16325C		C16256T		G16558A
U5b	C16294T		A16309G		C16266T		C16256T
	C16296T		C16256T				T16304C
	G16412C						
	G16496C						
	C16527T						
	G16526A						
	G16543A						
	A16318T						
	A16163G						
	C16186T						

**Table 4.** Population genetics comparison of Shina ethnic group with north-western Pakistani groups which mtDNA sequences data is available in Genbank, NCBI.

Populations	Sample size	Length of mtDNA	Polymorphic sites	RMP	POD	GD
Shina	09	16066–16569	26	0.0733	0.9266	1.0424
Kashmiri	09	16066–16569	21	0.0969	0.9031	1.0159
Kho	09	16066–16569	21	0.0938	0.9061	1.0194
Pathan	09	16066–16569	17	0.0742	0.9257	1.0414

RMP: random match probability; POD: power of discrimination*;* GD: genetic diversity.

The haplogroup ‘H’ was found predominant in Shina samples with a total frequency of 44.4%. This haplogroup ‘H’ is the most common European haplogroup that originated in Southwest Asia approximately 20,000–25,000 YBP. The haplogroup HV is the ancestral haplogroup of ‘H’. The HV and R0a are the descendant of haplogroup ‘R’ (Achilli et al. 2004). The haplogroup ‘U’ (i.e. a descendant of haplogroup ‘R’) was observed as major mtDNA haplogroup for Kashmiri samples in our analysis. The haplogroup ‘U’ possible place of origin is Western Asia around 46,500 years ago (Behar et al. 2012). It has different sub-clades U1–U9 which are generally conveyed crosswise over Northern and Eastern Europe, Central, Western and South Asia as well as North Africa (Hervella et al. 2016). The ‘HV’ is the ancestral haplogroup of ‘H’, i.e. observed as major haplogroup for Shina samples. The ‘HV’ is in turn a descendant of haplogroup ‘R’. Therefore, the haplogroup ‘R’ assumes as the ancestral haplogroup for both Shina and Kashmiri samples and this speculates a close genetic connection between Shina and Kashmiri populations. The shared mtDNA haplogroups (i.e. H2a and U7) between Shina and Kashmiri further reinforce this assumption.

**Figure 2.**
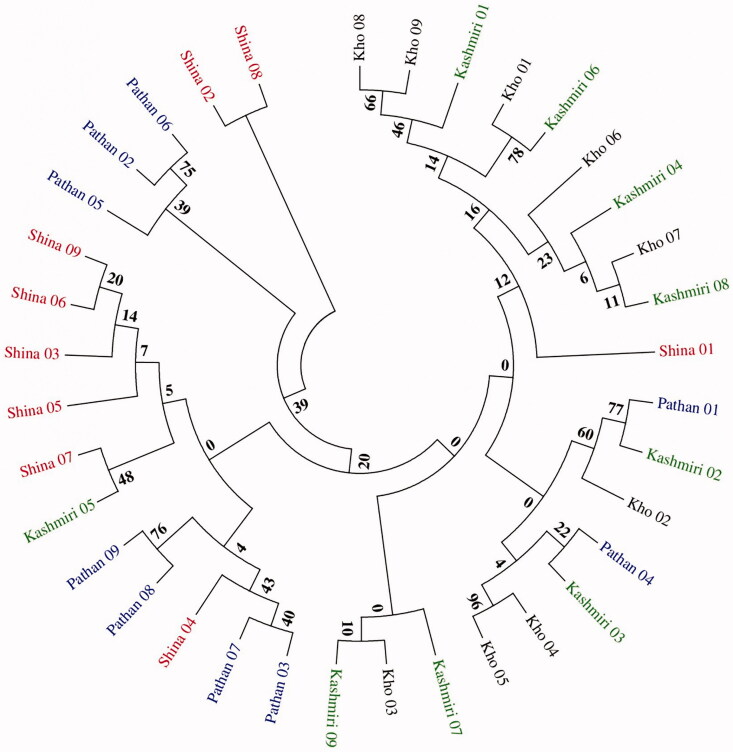
Phylogenetic tree analysis of the mtDNA D-loop region of Shina and surrounding north-western Pakistani populations.

During the neighbour-joining (NJ) and maximum-likelihood (ML)-based phylogenetic trees reconstruction the Shina and the rest of three surrounding north-western Pakistani populations’ samples formed separate major tree clades indicating their distinct genetic structure. The Shina samples were somehow found to share the phylogenetic tree clades with the Kashmiri in both MP and ML trees (Figure 2). Additional phylogenetic analyses were performed with mtDNA sequences of a European (Refugia) and Chinese (Han) population samples retrieved from Genbank, NCBI. However, similar clustering pattern was found for Shina sequences as observed in the previous analysis (Supplementary Material; Figures S2 and S3). The overall low confidence clustering pattern in these analyses mainly arose due to the small sample sizes of the population groups.

**Figure 3.**
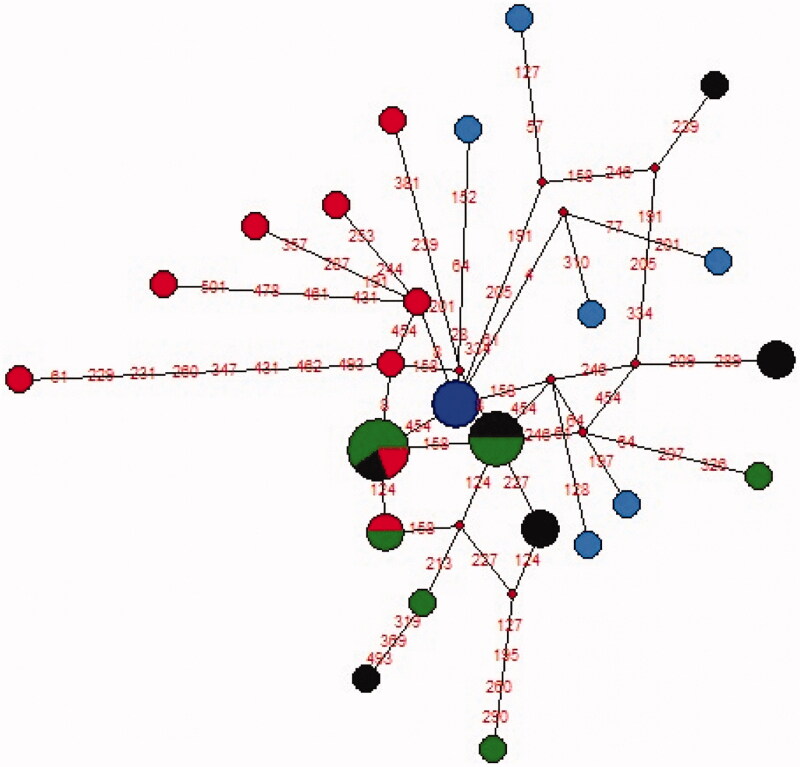
A phylogenetic network of Shina and other nearby north Western Pakistani populations. The haplotypes of each population in the figure are presented in different colours; Red: Shina, Green: Kashmiri, Blue: Pathan and Black: Kho. A node represents single mtDNA sequence and the size of each node depicts the haplotype frequency. The lines connecting the nodes depict the genetic relatedness among haplotypes.

We performed haplotypes networking analysis of Shina and the rest of available northern population groups’ samples. During this analysis, Shina samples were found to share two nodes with Kashmiri and single node with Kho population. The Kho and Kashmiri samples were also found to share few haplotype nodes. The close mtDNA based genetic relationship between these populations groups have previously been reported (Aziz et al. 2019). The Pathan samples were not clustered with Shina, Kho, and Kashmiri samples and marked clear distinction from these extreme northern groups (Figure 3).

## Discussion

The analysis of hypervariable sites in human mtDNA is commonly performed to investigate the population geographic ancestry (Mergen et al. 2004). The haplogroups and haplotypes composition stipulate the origin of individuals and populations. The predominant presence of Western Eurasian lineages in Shina samples revealed that gene flow to northern Pakistani territory happened from the West, i.e. Iran or from the North through Central Asia by the arrival of various trespassers in the past (McElreavey and Quintana-Murci 2005). The presence of a single South Asian haplogroup in Shina samples speculates the minor South Asian genetic component in the Shina. The few haplogroups and haplotypes sharing of Shina with nearby residing population groups inferred the limited genetic relationship of Shina with surrounding groups. The shared mtDNA haplogroups, common haplotypes nodes in haplotype network plot and close phylogenetic tree branches of Shina samples with Kashmiri represent a common maternal lineage between these populations. In few linguistic perspective and stratification studies about northern Pakistani groups, the core vocabulary and dialects of Shina and Kashmiri languages are presented and inferred that Shina dialects have been in contact and influenced by varying extent from Kashmiri and Persian (Schmidt and Kaul 2008). However, no close linguistic connection between Shina and Kho groups represented in these studies. The genetic relationship observed among these northernmost Pakistani population groups in the current study is in some way congruent to their anthropological and linguistic demographic reports.

## Conclusion

The investigation of the present study provided useful information about the genetic ancestry of Shina tribe residing in the remote northern region of Pakistan. The highest frequency of western mtDNA haplogroups in Shina samples indicates their West Eurasian ancestral origin. However, the limited presence of South Asian haplogroups reveal minor genetic admixture of Shina with surrounding South Asian groups. The comparative population genetic analyses divulged that the northernmost Pakistani population groups (i.e. Shina, Kho, and Kashmiri) residing in the Gilgit-Baltistan and close regions somehow shared their maternal lineages. However, the current study based on low sample size and the findings need to be validated with a larger sample size from Shina tribe. It would be noteworthy to plan genome-wide markers based population genetic investigation of Shina to better infer their genetic origin, demography and micro-evolutionary pattern shaped against the environmental burden.

## Supplementary Material

Supplemental Material
