# Supplementary material for: The genetic composition of Shina population from Gilgit-Baltistan, Pakistan based on mtDNA analyses
Source: Mitochondrial DNA B Resour. 2019 Oct 26;4(2):3802–8. doi: 10.1080/23802359.2019.1682474 (PMC7710323; doi:10.1080/23802359.2019.1682474)
Supplement: Supplemental Material [file TMDN_A_1682474_SM0070.zip › Asifullah Khan et al supplemental content.pdf]

SUPPLEMENTARY FIGURES

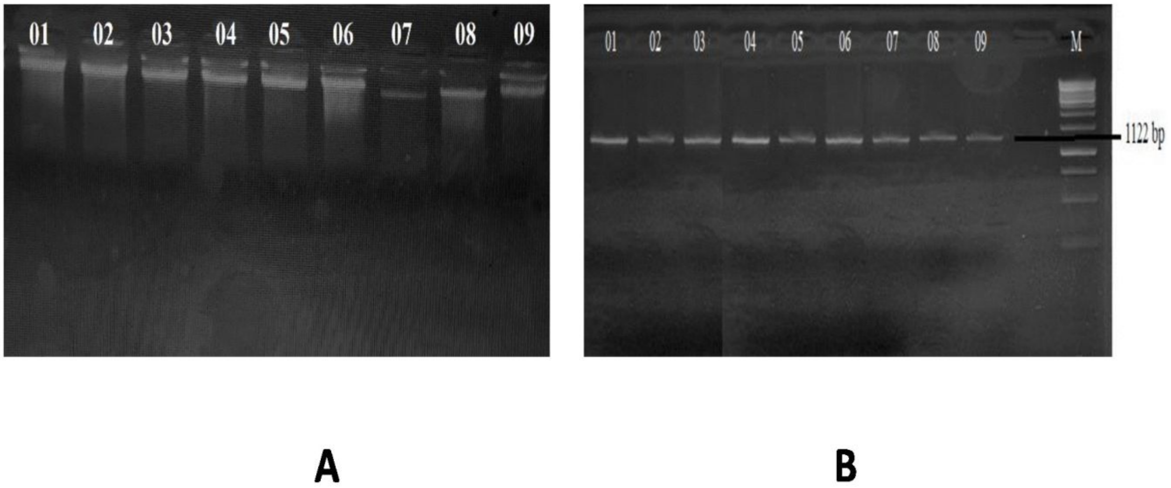

Figure S1: A, Agarose Gel Electrophoresis showing the bands of Genomic DNA. **B,** Agarose Gel Electrophoresis image of PCR amplified mtDNA HVRs of Shina samples. M, represents DNA ladder (1 Kb).

Peer Review Only

SUPPLEMENTARY MATERIAL

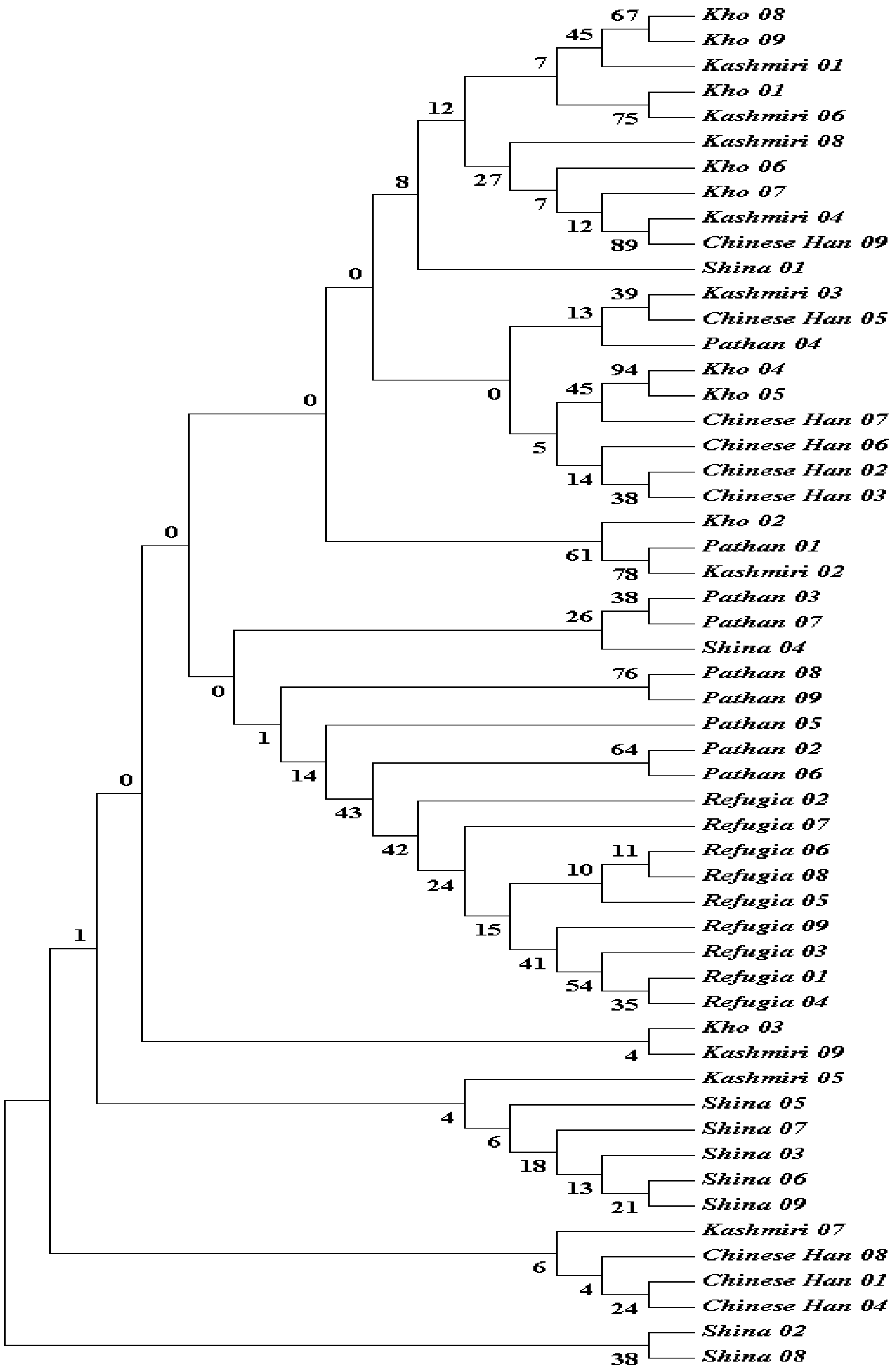

Figure S2: Neighbour Joining phylogenetic tree analysis of Shina with closely residing north-western Pakistani populations, European population (Refugia) and Chinese (Han) population.

SUPPLEMENTARY MATERIAL

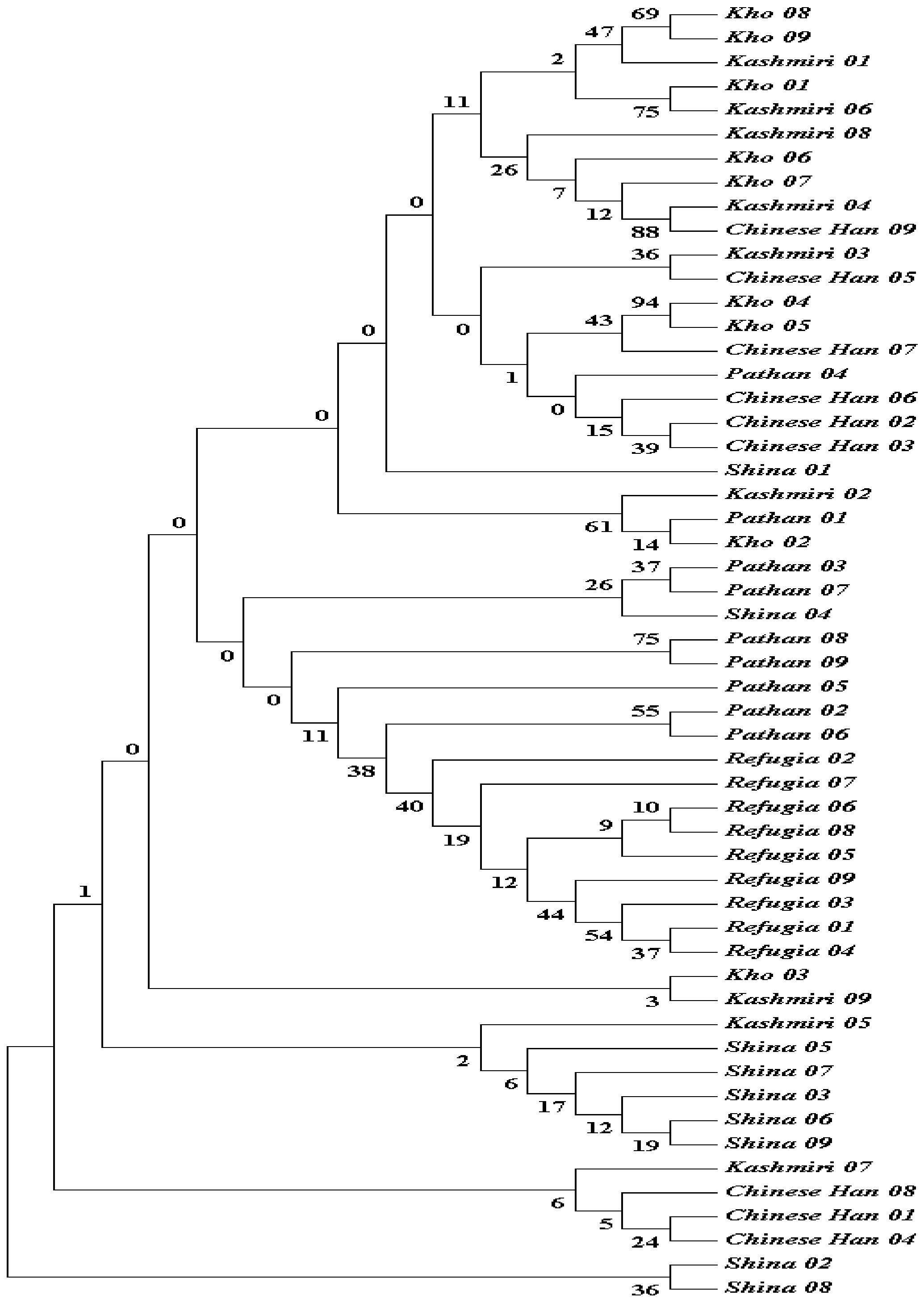

Figure S3: Maximum likelihood phylogenetic tree analysis of Shina with closely residing north-western Pakistani populations, European population (Refugia) and Chinese (Han) population.

SUPPLEMENTARY TABLES:

Table S1: Sample donor's data of Shina individuals from Gilgit Baltistan, Pakistan

SUPPLEMENTARY MATERIAL

Sample ID	Gender	Ethnic Group	Age	Resident district
**S-1**	Male	Shina	20	Roundo
**S-2**	Female	Shina	20	Gilgit
**S-3**	Male	Shina	20	Astore
**S-4**	Male	Shina	24	Roundo
**S-5**	Female	Shina	18	Gilgit
**S-6**	Male	Shina	21	Gilgit
**S-7**	Male	Shina	22	Gilgit
**S-8**	Male	Shina	21	Danyore
**S-9**	Male	Shina	21	Gilgit

Abbreviations: S; Shina

For Peer Review Only

SUPPLEMENTARY MATERIAL

Table S2: The accession numbers of the mtDNA sequences of north-western Pakistani ethnic groups (i.e. Kashmiri, Pathan and Kho) obtained from the National Center for Biotechnology Information (NCBI) for comparative analyses with Shina tribe

**Population**	**Accession Numbers (Genbank, NCBI)**
Kashmiri	KX084271, KX084272, KX084079, KX084069, KX084076, KX084103, KX084100, KX084370, KX084242
Kho	MK124589, MK124590, MK124591, MK124592, KM124593, KM124599, KM124600, KM124603, KM124604
Pathan	KT375290, KT375288, KT375285, KT375279, KT375293, KT375292, KT375291, KT375297, KT375289

For Peer Review Only

SUPPLEMENTARY MATERIAL

Table S3: Position of mutations in mtDNA of Shina population in comparison with rCRS

**Transitions**	**Position 16066-16569**	**Mutation (%)**
**A-G**	2	7.4%
**G-A**	4	15%
**C-T**	9	33%
**T-C**	8	30%
**Total**	23	85%
**Transversion**	–	–
**G-T**	0	0
**G-C**	3	11%
**A-T**	1	4%
**T-A**	0	0
**Total**	4	15%
**Total**	27	100%

For Peer Review Only
